# Psychedelic experience dose-dependently modulated by cannabis: results of a prospective online survey

**DOI:** 10.1007/s00213-021-05999-1

**Published:** 2021-11-04

**Authors:** Joanna Kuc, Hannes Kettner, Fernando Rosas, David Erritzoe, Eline Haijen, Mendel Kaelen, David Nutt, Robin L. Carhart-Harris

**Affiliations:** Department of Brain Sciences, Faculty of Medicine, Centre for Psychedelic Research, Imperial College London, London, W12 0NN UK

**Keywords:** Psychedelics, Cannabis, Subjective experience, Set and setting, Mystical experience, Challenging experience, Peak experience, Recreational use, Harm reduction

## Abstract

**Rationale.:**

Classic psychedelics are currently being studied as novel treatments for a range of psychiatric disorders. However, research on how psychedelics interact with other psychoactive substances remains scarce.

**Objectives:**

The current study aimed to explore the subjective effects of psychedelics when used alongside cannabis.

**Methods:**

Participants (*n* = 321) completed a set of online surveys at 2 time points: 7 days before, and 1 day after a planned experience with a serotonergic psychedelic. The collected data included demographics, environmental factors (so-called setting) and five validated questionnaires: Mystical Experience Questionnaire (MEQ), visual subscales of Altered States of Consciousness Questionnaire (ASC-Vis), Challenging Experience Questionnaire (CEQ), Ego Dissolution Inventory (EDI) and Emotional Breakthrough Inventory (EBI). Participants were grouped according to whether they had reported using no cannabis (*n* = 195) or low (*n* = 53), medium (*n* = 45) or high (*n* = 28) dose, directly concomitant with the psychedelic. Multivariate analysis of covariance (MANCOVA) and contrasts was used to analyse differences in subjective effects between groups while controlling for potential confounding contextual ‘setting’ variables.

**Results:**

The simultaneous use of cannabis together with classic serotonergic psychedelics was associated with more intense psychedelic experience across a range of measures: a linear relationship was found between dose and MEQ, ASC-Vis and EDI scores, while a quadratic relationship was found for CEQ scores. No relationship was found between the dose of cannabis and the EBI.

**Conclusions:**

Results imply a possible interaction between the cannabis and psychedelic on acute subjective experiences; however, design limitations hamper our ability to draw firm inferences on directions of causality and the clinical implications of any such interactions.

**Supplementary Information:**

The online version contains supplementary material available at 10.1007/s00213-021-05999-1.

## Introduction

Psychedelics are now being extensively researched with respect to their potential usage in clinical settings as an addition to psychotherapy for treating various mental health disorders. A growing body of evidence suggests their efficacy for addressing conditions such as depression (Carhart-Harris et al. 2016, 2018a), depressive and anxiety symptoms in patients with terminal cancer (Griffiths et al. 2006, 2016; Ross et al. 2016), obsessive–compulsive disorder (OCD) (Moreno et al. 2006) or addiction (Johnson et al. 2014; Bogenschutz et al. 2015). These studies suggest that critical mediators of the efficacy of psychedelic-assisted psychotherapies lie in the acutely experienced psychedelic state (Roseman et al. 2018).

**Fig. 1:**
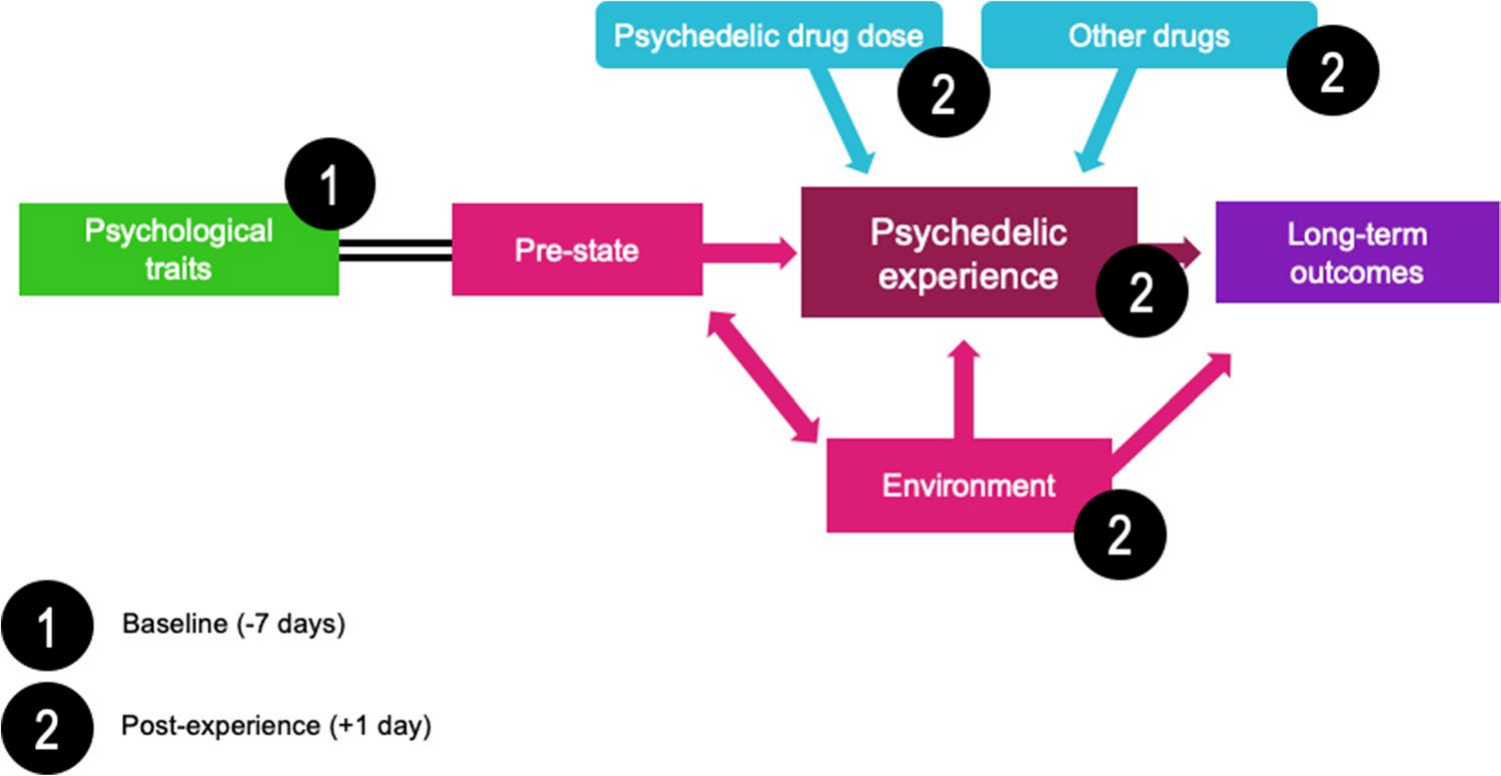
Timepoints of the conducted online surveys in relation to when the psychedelic experience took place. First set of surveys was filled at Timepoint 1 (baseline) which took place 7 days before taking the psychedelic, while the second set of questionnaires was filled at Timepoint 2 (post-experience), one day after the psychedelic experience. ‘Extra-pharmacological’ factors can change the course and effects of the psychedelic experience. Psychological traits refer to specific personality traits that can predispose an individual to a certain type of experience; for example, trait ‘absorption’ promotes intensity of the psychedelic experience (Studerus et al. 2012; Haijen et al. 2018) Polygenic contributions to personality seem likely but are not yet reliably defined. However, some interesting and relevant candidates have been identified (Ott 2007). Pre-state can also be described as ‘set;’ it indicates the pre-experience mindset like level of anxiety, expectations and intentions; it describes the readiness to ‘surrender’ to the effects of the drug (Russ and Elliott 2017). Psychedelic experience describes the features of the acute psychedelic experience; these can be measured through subjective rating scales (Haijen et al. 2018; Roseman et al. 2018) or brain imaging (Carhart-Harris et al. 2016; Madsen et al. 2019). Environment can also be described as setting; it refers to the physical surrounding and various environmental influences. Dose relates to the drug dosage, which can be crucial in defining the course of the experience (Griffiths et al. 2011; Studerus et al. 2012). Other drugs refer to drugs taken simultaneously with the psychedelic and alter the subjective experience. This variable is a key focus of this study. Long-term outcomes can relate to a number of variables, such as symptom severity of a psychiatric condition or changes in personality traits. Adapted from: Carhart-Harris et al. (2018a, b)

Serotonergic ‘classic’ psychedelics (i.e., psychedelics with some direct serotonin 2A receptor agonist properties) are known to be capable of producing profound distortions in perceptual processes, mood and cognition (Halberstadt and Geyer 2011). Psychedelics are often associated with alterations in visual and other sensory perception (perceptual intensification, illusion, mental imagery, elementary and complex imagery), as well as synaesthesia (Studerus et al. 2010). They also induce emotional effects, such as general intensification of feelings, increased access to emotions and cognitive effects, defined by changes to the normal flow of cognition (Swanson 2018). Additionally, several controlled studies reported occurrences of ‘mystical-type experiences’ under psychedelics (feelings of unity, transcendence of time and space, deeply felt positive mood, alleged ineffability and sacredness), which may be important mediators of treatment responses (Maslow 1959; Stace 1960; Griffiths et al. 2011; Garcia-Romeu et al. 2014; Nichols 2016; Roseman et al. 2019). Although the dose of the psychedelic is crucial in defining the course of a psychedelic experience (Griffiths et al. 2011; Studerus et al. 2012), extrapharmacological factors such as ‘set’ and ‘setting’ (Fig. 1) also play a key role in determining the subjective effects. For an in-depth analysis of predictors of psychedelic response, please refer to the study by Haijen et al. (2018).

### Use of psychedelics with other substances

Outside clinical settings, psychedelics are commonly used concomitantly with other psychoactive substances (Grov et al. 2009; Licht et al. 2012): ranging from cannabis smoked together with ayahuasca during ceremonies of the Brazilian Santo Daime Church (MacRae 1998), to lysergic acid diethylamide (LSD) taken with 3,4-Methylenedioxymethamphetamine (MDMA) at music festivals—referred to as ‘candyflipping’ (Schechter 1998; Grov et al. 2009; Licht et al. 2012; Chary et al. 2018). Case reports show that users tend to combine psychoactive substances to maximise effects they consider to be positive (e.g. euphoria), and minimise negative effects, such as dysphoria or unwanted somatic symptoms (Chary et al. 2018). However, the studies that assess the subjective experience of concomitant drug use quantitatively are limited, so to address this gap in the literature, we investigated the interaction between psychedelics and cannabis, a substance reported to be commonly used alongside LSD (Boys 2001; Grov et al. 2009).

The two key chemical constituents of cannabis are Δ-9 tetrahydrocannabinol (THC) and cannabidiol (CBD), and while most of their effects are believed to be exerted through cannabinoid type 1 (CB1) and type 2 (CB2) receptors, several studies have also suggested they interact with serotonin (5-HT) receptors. Previous animal work reports that CBD facilitates 5-H_1A_ receptor-mediated neurotransmission (Resstel et al. 2009) which can lead to an increase in 5-HT and glutamate levels, which in turn translates to antidepressant-like effects (Linge et al. 2016). Recent work in humans corroborates this finding and shows that at high concentrations, CBD acts as an inverse agonist on 5-HT_1A_ receptors (Martínez-Aguirre et al. 2020). Additionally, chronic exposure to THC has also been reported to promote pro-hallucinogenic signalling of 5-HT_2A_R in mice; however, this has not yet been studied during acute exposure or in humans (Ibarra-Lecue et al. 2018). In any case, due to the overlap in receptor targets, we can expect a possible synergistic interaction between serotonergic psychedelics and cannabis.

Clarifying interactions between psychedelics and cannabis might bring insights which could have important consequences for psychedelic-assisted clinical theory, for example: to assess the initial potential to use psychedelics and cannabis in tandem to maximise beneficial effects of both, or initiate a conversation on guidelines regarding the required abstinence period before being admitted to a clinical study. Additionally, given the widespread simultaneous use of psychedelics with other substances (Boys 2001; Grov et al. 2009; Licht et al. 2012), the present investigation might have value for harm reduction messaging, providing data on previously unreported potential side effects of this polydrug combination. Finally, attaining a better understanding of how these drugs interact on a subjective level through a self-report study may allow for developing hypotheses to be tested in controlled laboratory studies, which in turn can provide novel insights into potential biological interactions, e.g. at the pharmacological level.

## Methods

This study was approved by the Imperial Research Ethics Committee and the Joint Research Compliance Office at Imperial College London. Study IREC reference: 17IC3746.

### Design

A software platform (https://www.psychedelicsurvey.com/) was used that enables researchers to collect a large amount of data on different ways of taking psychedelics in a non-controlled, naturalistic and observational manner.^1^ The platform was designed to recruit adults, who already had an intention of taking a psychedelic and ask them to fill out several surveys at specified time points. The surveys were sent to participants through automatically generated emails sent out at specific time points depending on the date the participant planned their experience to take place. The inclusion criteria for the survey participants were as follows: at least 18 years old, a good understanding of the English language, and having the intention to take a serotonergic psychedelic drug (psilocybin/magic mushrooms/truffles, LSD/ 1-propionyl-lysergic acid diethylamide (1P-LSD), ayahuasca, *N*,*N*-Dimethyltryptamine (DMT), 5-methoxy-*N*,*N*-dimethyltryptamine (5-MeO-DMT), mescaline, 2,5-dimethoxy-4-bromophenethylamine (2C-B) or others with a similar mechanism of action). In the current study, data from 321 participants (*n* = 321) was used. For a full overview of the study and the sample, please refer to Haijen et al. 2018.

The initial design includes 5 time points (Haijen et al. 2018); however, for simplicity and focus, the current study included just two of the data collection time points, illustrated in Fig. 1. The baseline time point (1) took place 7 days before the planned experience. Collected data included the following demographics: age, sex, nationality, education level, employment status, history of psychiatric conditions and history of drug use. The post-experience time point (2) was collected one day after the subject's psychedelic experience. In this survey, collected data included drug variables (the type of psychedelic used, other drugs used, dose); Mystical Experience Questionnaire (MEQ), visual subscales of the Altered States of Consciousness Rating Scale (ASC-Vis), Challenging Experience Questionnaire (CEQ), Ego Dissolution Inventory (EDI) and Emotional Breakthrough Inventory (EBI) questionnaires, as well as the information about the setting in which the experience took place in (see below).

### Drug usage

As a part of the post-experience survey, participants specified the psychedelic they used from one of the following options: Psilocybin/magic mushrooms/truffles; LSD/1P-LSD; Ayahuasca; DMT/5-MeO-DMT; Mescaline (Peyote, San Pedro). Additionally, participants were asked to indicate the (total) dose they used, choosing between the following options: a low dose (≦50 μg of LSD), a moderate dose (≦100 μg of LSD), a high dose (≦200 μg of LSD), a very high dose (≦300 μg of LSD) or an extremely high dose (> 300 μg of LSD). This approach implemented in the following (Nour et al. 2016; Roseman et al. 2019) enables a standardised dose account that allows comparisons across different psychedelics. Please note that this was not a calculation, but a subjective question based on perceived quantity of a drug by the user. The answers provided by the participants were then recoded into numerical Likert-scale 1–5 values, where 1 was equal to a low dose and 5 an extremely high dose of a psychedelic.

**Fig. 2:**
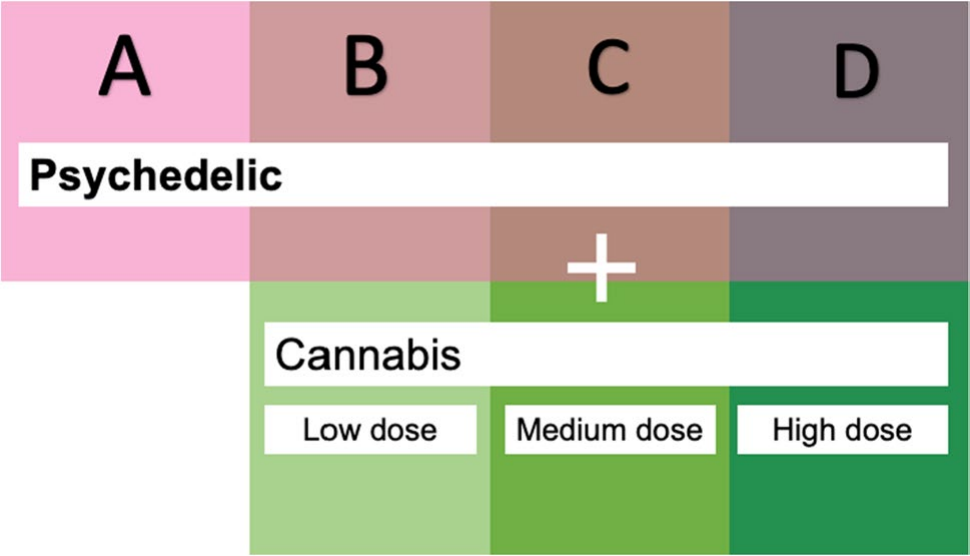
Criteria for sample grouping. ‘Group A’ is composed of participants who only used a psychedelic drug during the experience; ‘Group B’—a psychedelic with cannabis at low dose; ‘Group C’—a psychedelic with cannabis at medium dose; ‘Group D’—a psychedelic with cannabis at high dose. Note that the dose of the psychedelic was not specified in this model. The dose of cannabis was rated subjectively by the participant

Survey participants were also asked to indicate if they used other types of drugs (cannabis, alcohol, stimulants, tobacco) during their psychedelic experience. For this, they had to choose one of the following options for each of the listed drugs: ‘I have not used the drug during experience’; ‘low dose’; ‘medium dose’; ‘high dose’. While not specifying the exact dose, this subjective-self report allows estimating how strongly have the participants felt the effects of the drug. For the purpose of the current study, participants were grouped based on their simultaneous use of psychedelics and cannabis, as represented by Fig. 2. The answers provided by the participants were then recoded into numerical Likert-scale 0–3 values, where 0 was equal to none and 3 to high dose of cannabis.

### Setting, framework and environmental factors

Participants were asked questions about the ‘setting’ (i.e., therapeutic, recreational, retreat) and guiding framework (i.e., spiritual, religious, psychotherapeutic, shamanic and others). For details, see Appendix 1. The purpose of collecting this data was to analyse it as potential confounders (please see the ‘14’ section for more details on identifying confounders).

### Subjective experience questionnaires

In the current study, five of the measures frequently used to analyse the effects of psychedelics were employed, based upon the idea to represent a broad, comprehensive spectrum of the subjective effects reported on psychedelics.

#### Mystical Experience Questionnaire

The Mystical Experience Questionnaire (MEQ) (Pahnke and Richards 1970; Pahnke et al. 1971) includes 30 items (for details, see Table 9, Appendix 2) rated on a six-point Likert scale (0–5). The total MEQ score was calculated as the average of all 30 items and multiplied by 20 to provide a value in a 0–100 range (MacLean et al. 2012). The MEQ was previously used in both non-clinical (Haijen et al. 2018; Roseman et al. 2019; Kettner et al. 2021) and clinical work (Barrett et al. 2015; Liechti et al. 2017; Schmid et al. 2021; Stenbæk et al. 2021; Stauffer et al. 2021).

#### Visual subscales of the Altered States of Consciousness Questionnaire

The ‘Elementary Imagery’, ‘Complex Imagery’ and ‘Audio-Visual Synaesthesia’ subscales of the ASC include 9 questions (Studerus et al. 2010); (for details, see Table 10, Appendix 2). The answers to ASC-Vis were given using a visual analogue scale (VAS), with 0 defined as ‘No, not more than usually’, and 100 defined as ‘Yes, entirely or completely’ and the total score calculated as the sum of all 9 items (Roseman et al. 2019). The ASC was previously used in non-clinical (Heink et al. 2017; Haijen et al. 2018; Roseman et al. 2019; Kettner et al. 2021) and clinical work (Liechti et al. 2017; Roseman et al. 2018; Schmid et al. 2021). In the current study, only the visual subscales of ASC are used, which was previously done by Kettner et al. 2021.

#### ***Challenging Experience Questionnaire***

The Challenging Experience Questionnaire (CEQ) (Barrett et al. 2016) consists of 26 questions (for details, see Table 11, Appendix 2). The participants were asked to provide an answer to each of the questions using a six-point Likert scale format (0–5). The total CEQ score was calculated as the average of all 26 items and multiplied by 20 to provide a value in a 0–100 range (Roseman et al. 2019). The CEQ was previously used in non-clinical (Nour et al. 2016; Haijen et al. 2018; Roseman et al. 2019; Kettner et al. 2021) and clinical work (Stauffer et al. 2021).

#### Ego Dissolution Inventory

The Ego Dissolution Inventory (EDI) (Nour et al. 2016) consists of 8 questions rated on a 0–100 VAS (for details, see Table 12, Appendix 2). The total EDI score was calculated by taking an average out of the 8 items. The EDI was previously used in non-clinical work (Nour et al. 2016, 2017; Haijen et al. 2018).

#### Emotional Breakthrough Inventory

The Emotional Breakthrough Inventory (EBI) (Roseman et al. 2019) consists of 6 items (for details, see Table 13, Appendix 2) rated on a 0–100 VAS. The total score was calculated as the average of the 6 items. The EBI was previously used in non-clinical work (Haijen et al. 2018; Roseman et al. 2019; Kettner et al. 2021).

### Statistical analysis

Responses were recoded as numerical values, and the subscale and total scores for each of the questionnaires were computed. Listwise deletion was performed for cases where participants have not completed all of the surveys at specified time points and only entries from participants who completed all of the required surveys were included in the analyses. The statistical analyses performed over the arranged data are described in the following.

#### Multivariate analysis of covariance

The samples were grouped as represented by Fig. 2. The confounding factors in the current study were selected based on exploratory distribution of variables which, based on the previous literature, have been suggested to act as potential confounders (Haijen et al. 2018). To identify potential confounding factors between the resulting groups of cannabis use, ANOVAs were performed using subjectively described cannabis dose (none, medium, low or high) as an independent variable, and the following as dependent variables: recoded values (1–5) representative of a dose of the psychedelic, relevant elements of setting, personality questionnaires and psychiatric illnesses. Variables where the difference in distribution between the groups of cannabis users was significant (*p* < 0.05) were classified as potential confounding factors. The non-normal distribution of variables was not considered to be problematic, in accordance with literature suggesting that the violation of the assumption of parametric distribution might not be problematic for the quality of final output in regular linear models, especially at high sample rates (Schmider et al. 2010; Blanca et al. 2017).

Pearson correlation coefficients were calculated to identify multicollinearity among the chosen confounders; confounders with moderate correlations (*r* > 0.4) were addressed through selective exclusion. To confirm the absence of multicollinearity, separate linear regressions with each of the confounders as a dependent variable and the remaining confounders as independent variables were constructed. The variance inflation factor (VIF) cut-off point set to 5. All VIF points were less than 5, indicating that multicollinearity was not a concern.

The multivariate analysis of covariance (MANCOVA) analysis was performed using MEQ, Altered States of Consciousness Questionnaire (ASC-Vis), CEQ, EDI and EBI scores as the dependent variables, while the dose of cannabis (none, low, medium or high) was used as a fixed factor. To allow comparison across models, *z* score standardisation was performed on all included variables. The group using a psychedelic without cannabis was used as a reference group. Pillai’s trace was chosen as a test statistic due to its robustness against violations of MANCOVA assumptions, such as multivariate normality (Olson 1974). Parameter estimates (i.e. beta values) together with their effect sizes (Sullivan and Feinn 2012) were analysed, and their estimated marginal means were calculated. Post hoc Bonferroni’s correction was used to control for multiple comparisons. To compare the trends across the experimental groups, polynomial contrasts were used at three degrees: linear, quadratic and cubic. Where *p* < 0.05, the relationship was accepted as significant.

#### Regression modelling

To further investigate the psychological effects of cannabis dose, we computed linear, quadratic and cubic regression models for each of the questionnaire outcomes using dose (quantified as none = 0, low = 1, medium = 2 and high = 3) as an independent variable, treating it as continuous. The application of linear regression to ordinal independent variables was chosen given the presumed continuous structure of the underlying variable (dosage), as well as the large sample size, conditions which favour the robustness of metric models with ordinal regressors. All models included setting elements ‘Party’, ‘Shamanic’, ‘Singing’ and ‘Disruptions’ as covariates. Model selection between models was then conducted using the Bayesian Information Criterion (BIC). Only the model with the lowest BIC is reported.

To allow for a more detailed understanding, the means for all subscales of questionnaires with significant correlations were also represented in a form of radar charts.

### Software

Statistical analyses were conducted IBM SPSS Statistics for Windows, Version 25.0 (IBM Corp.); MATLAB Release 2018b (The MathWorks, 2018), RStudio V1.2.1335 (Rstudio Inc., 2019), GraphPad Prism version 8.00 (GraphPad Software, 2018) and Microsoft Office 365 package (Microsoft, 2011).

## Results

### Demographics

While 654 participants have signed up for the survey, 321 of them have completed all of the time points analysed in the current study (baseline, pre-experience and post-experience); thus, only this subset has been included for the final analysis. The average age of the survey participants (*n* = 321) was 30.6 ± 11 years (mean ± SD), with males representing 68.8% of the sample (see Table 1). For a more detailed description, see the ‘18’ section (supplementary material).

**Table 1:** Demographics data for the survey participants. Values represented in the table are absolute frequencies and numbers in brackets are the percentage values

	(n = 321)	%
Gender		
Male	221	68.8
Female	79	24.6
*Blank*	21	6.5
Age	30.6 ± 11	
Nationality		
US—United States	83	25.9
GB—United Kingdom	57	17.8
DK—Denmark	38	11.8
DE—Germany	17	5.3
CA—Canada	13	4
AU—Australia	9	2.8
NL—Netherlands	6	1.9
NO—Norway	6	1.9
FI—Finland	6	1.9
IE—Ireland	5	1.6
Other countries (30 countries)	60	18.1
*Blank*	21	6.5
Education level		
Bachelor’s degree (or equivalent)	106	33
Post-graduate degree (e.g. masters or doctorate)	69	21.5
Some university (or equivalent)	56	17.4
High school diploma/A-level education (in UK)	42	13.1
Some high school/GCSE level (in UK)	22	6.9
Left school before age 16 without qualification	5	1.6
*Blank*	21	6.5
Employment status		
Full-time job	113	35.2
Student	103	32.1
Part-time job	49	15.3
Unemployed	29	9
Retired	6	1.9
*Blank*	21	6.5
Psychiatric history		
No psychiatric disorder	205	63.9
Anxiety	53	16.5
Major depressive disorder	53	16.5
ADHD	14	4.4
Personality disorder	12	3.7
Substance abuse disorder	10	3.1
Bipolar disorder	8	2.5
Eating disorder	7	2.2
OCD	6	1.9
Alcohol dependence	3	0.9
Hallucinogen persisting perception disorder	2	0.6
Psychotic disorder	2	0.6
Schizophrenia	1	0.3

The values represent mean age (± SD) or otherwise absolute frequencies together with percentage

ADHD attention deficit hyperactivity disorder

OCD obsessive compulsive disorder

### Drug choices

The most commonly used psychedelic was LSD (50.2%), followed by psilocybin (29.3%), which includes any psilocybin-containing substances, e.g. magic mushrooms, or truffles. Other reported serotonergic psychedelics included the following: ayahuasca (11.8%), DMT (3.4%), mescaline (1.9%) and 2C-B (1.2%), synthetic mescaline-HCl and 2C-B-HCl (0.3%), 4-(2-fluoroethylthio)-2,5-dimethoxyphenethylamine (2C-T-21) (0.3%), O-Acetylpsilocin (4-AcO-DMT) (0.3%), 4-hydroxy-*N*-methyl-*N*-ethyltryptamine (4-Ho-MET) (0.3%) and 6-allyl-6-nor-LSD (AL-LAD) (0.3%), psilocybin in combination with mescaline (0.3%) and psilocybin in combination with Syrian Rue (0.3%).

**Fig. 3:**
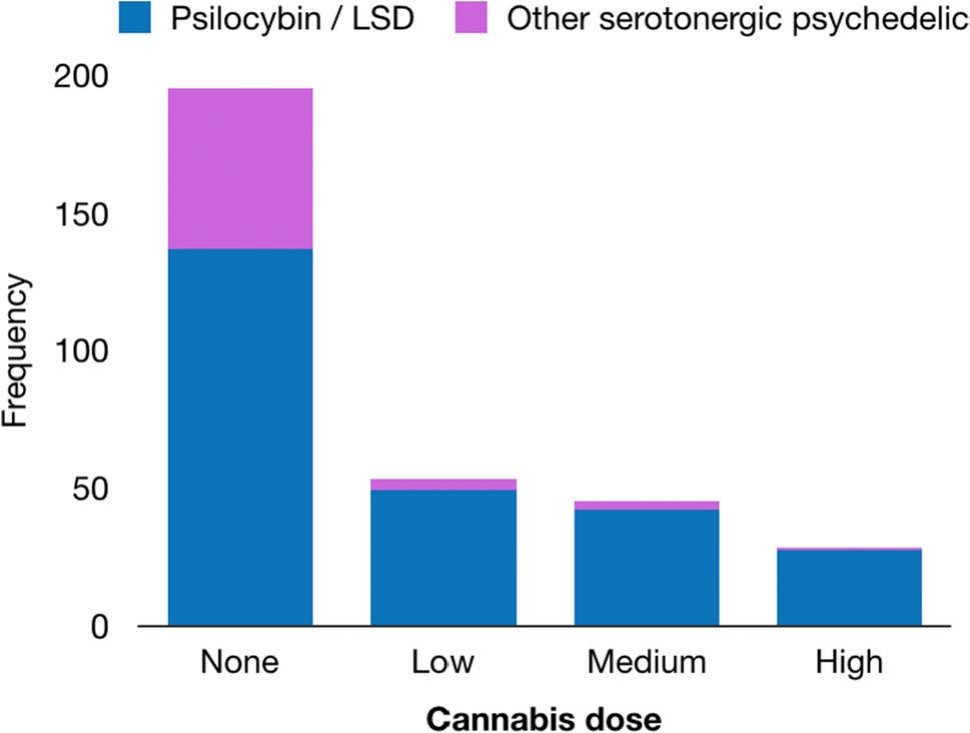
Absolute frequencies of drug choices among the survey participants (*n* = 321) grouped based on the used cannabis dose (none: *n* = 195; low: *n* = 53; medium: *n* = 45; high: *n* = 28). Those who used LSD or psilocybin are shown in blue, while those who used other serotonergic psychedelics in purple

Overall, (Fig. 3), 39.3% reported using cannabis during the psychedelic experience. Most frequently, it was used at low doses (16.5%), followed by medium (14.0%) and high (8.7%). Psilocybin and LSD were most often used across all the groups. The highest frequency of other serotonergic psychedelics was reported for the group not using cannabis (29.7%), and subsequently decreased with increasing the cannabis dose: 7.5% at low, 6.7% at medium and 3.6% at high cannabis dose.

Across the whole sample, 10.6% of participants used alcohol, 1.9% used stimulants and 25% used tobacco. Out of those who used no cannabis, 93.8% used no alcohol, 4.6% used a low dose of alcohol, 1.5% used a medium dose of alcohol and none (0%) used a high dose of alcohol; 99.5% used no stimulants, 0.5% used a low dose of stimulants and none (0%) used a medium or high dose of stimulants; 85.6% used no tobacco, 6.7% used a low dose of tobacco, 6.7% used a medium dose of tobacco and 1% used a high dose of tobacco. Among those who used a low cannabis dose, 84.9% used no alcohol, 11.3% used a low dose of alcohol, 3.8% used a medium dose of alcohol and none (0%) used a high dose of alcohol; 98.1% used no stimulants, 1.9% used a low dose of stimulants and none (0%) used a medium or high dose of stimulants; 64.2% used no tobacco, 28.3% used a low dose of tobacco, 5.7% used a medium dose of tobacco and 1.9% used a high dose of tobacco. In the medium cannabis dose group, 86.7% used no alcohol, 8.9% used a low dose of alcohol, 2.2% used a medium dose of alcohol and 2.2% used a high dose of alcohol; 93.3% used no stimulants, 2.2% used a low dose of stimulants, 4.4% used a medium dose of stimulants and none (0%) used a high dose of stimulants; 55.6% used no tobacco, 31.1% used a low dose of tobacco, 11.1% used a medium dose of tobacco and none (0%) used a high dose of tobacco. In the high cannabis dose group, 71.4% used no alcohol, 21.4% used a low dose of alcohol, 3.6% used a medium dose of alcohol and 3,6% used a high dose of alcohol; 96.4% used no stimulants, none (0%) used a low dose of stimulants, 3.6% used a medium dose of stimulants and none (0%) used a high dose of stimulants; 50% used no tobacco, 21.4% used a low dose of tobacco, 17.9% used a medium dose of tobacco and none (0%) used a high dose of tobacco. For absolute frequencies, please see Table 9 (Supplementary material).

### Identification of potential confounding variables

One-way ANOVA tests were used to identify variables that were significantly correlated with cannabis dose, so they could be used later for controlling potential confounding effects. Using univariate ANOVAs, it was found that retreat setting (*p* = 0.001), party setting (*p* = 0.001), shamanic framework (*p* = 0.018), live singing/chanting (*p* = 0.007) and disruptions (*p* = 0.018) significantly differed across amounts of cannabis use in addition to the psychedelic (Table 2). These variables were therefore included in further analyses. Please note that the analysis showed that dose of the psychedelic (for details, see the ‘5’ section) was distributed equally across the different cannabis conditions; therefore, it was not classified as covariates and excluded from further analysis.

**Table 2:** Distribution of covariates across different conditions of cannabis use (none, low, medium and high dose) together with a psychedelic

		Dose of cannabis									
		None		Low		Medium		High			
		*x̄*	*σ*	*x̄*	*σ*	*x̄*	*σ*	*x̄*	*σ*	F	Sig
	Dose of psychedelic	2.60	0.95	2.76	1.01	2.80	1.02	2.68	1.11	1.208	0.306
Setting	Retreat	0.19	0.40	0.05	0.23	0.12	0.33	0.00	0.00	5.317	0.001 **
	Therapeutic	0.41	0.49	0.44	0.50	0.47	0.50	0.27	0.45	1.177	0.318
	Party	0.17	0.38	0.29	0.46	0.12	0.33	0.42	0.50	5.817	0.001 **
Framework	Spiritual	0.47	0.50	0.48	0.51	0.44	0.50	0.39	0.50	0.240	0.868
	Religious	0.10	0.30	0.06	0.24	0.05	0.21	0.04	0.19	1.006	0.390
	Psychotherapeutic	0.28	0.45	0.28	0.46	0.32	0.47	0.29	0.46	0.073	0.975
	Shamanic	0.29	0.46	0.27	0.45	0.18	0.39	0.04	0.19	3.416	0.018 *
	Live singing / chanting	0.31	0.46	0.25	0.44	0.16	0.37	0.04	0.19	4.073	0.007 **
	Music	0.84	0.37	0.87	0.35	0.86	0.35	0.79	0.42	0.347	0.792
	Emotional support	0.54	0.50	0.48	0.51	0.52	0.51	0.61	0.50	0.421	0.738
	Strangers	0.29	0.46	0.33	0.47	0.25	0.44	0.39	0.50	0.641	0.589
	Disruptions	0.22	0.42	0.25	0.44	0.27	0.45	0.5	0.51	3.401	0.018 *
	Nature	0.60	0.49	0.65	0.48	0.68	0.47	0.79	0.42	1.474	0.222
	Comfortable furniture	0.74	0.44	0.77	0.43	0.80	0.41	0.82	0.39	0.410	0.746
	Noise	0.24	0.43	0.23	0.43	0.23	0.42	0.32	0.48	0.357	0.784
	Threat	0.06	0.24	0.08	0.27	0.07	0.26	0.14	0.36	0.870	0.457
Personality	MODTAS	43.23	17.56	45.33	15.62	41.64	16.78	40.44	17.65	0.608	0.610
	(TIPI) extraversion	7.47	3.27	7.36	3.39	7.66	2.78	7.43	3.44	0.077	0.973
	(TIPI) agreeableness	9.74	2.45	9.55	2.26	9.43	2.07	9.68	2.96	0.255	0.858
	(TIPI) conscientiousness	9.53	2.77	9.36	3.05	9.90	3.12	8.25	2.91	2.051	0.107
	TIPI emotional stability	9.32	3.18	9.97	3.10	9.36	3.01	9.21	3.19	0.687	0.561
	TIPI openness	11.75	1.89	11.88	1.84	11.77	2.20	11.79	2.35	0.062	0.980
Mental illness	Major depressive disorder	0.22	0.42	0.15	0.36	0.34	0.48	0.24	0.44	1.837	0.140
	Bipolar disorder	0.03	0.18	0.07	0.25	0.03	0.18	0.04	0.21	0.545	0.652
	Schizophrenia	0.01	0.07	0.02	0.13	0.00	0.00	0.00	0.00	0.475	0.700
	Anxiety	0.24	0.43	0.21	0.41	0.35	0.48	0.12	0.33	1.908	0.128
	Substance abuse disorder	0.03	0.18	0.02	0.13	0.06	0.24	0.04	0.21	0.424	0.736
	Alcohol dependence	0.02	0.14	0.02	0.13	0.03	0.18	0.04	0.21	0.201	0.896
	HPPD	0.01	0.10	0.04	0.19	0.00	0.00	0.00	0.00	0.942	0.421
	Psychotic disorder	0.01	0.10	0.02	0.13	0.00	0.00	0.00	0.00	0.291	0.832
	Personality disorder	0.02	0.14	0.05	0.22	0.09	0.29	0.08	0.28	1.763	0.154
	ADHD	0.07	0.25	0.15	0.36	0.14	0.35	0.12	0.33	1.860	0.136
	OCD	0.02	0.13	0.04	0.19	0.09	0.29	0.00	0.00	2.211	0.087
	Eating disorder	0.07	0.25	0.04	0.19	0.03	0.18	0.04	0.21	0.422	0.737
Meds	Ever treated with medication	0.31	0.46	0.39	0.50	0.33	0.48	0.50	0.52	0.827	0.481
	Currently on any medication	0.16	0.37	0.30	0.47	0.33	0.49	0.22	0.44	1.141	0.336
	Currently on antidepressants	0.03	0.17	0.04	0.19	0.07	0.26	0.02	0.16	1.027	0.380

Values indicate means (+ − SD)

^**^ = *p* < 0.01

*x̄* mean

*σ* standard dev

A correlation matrix among the selected covariates was constructed to test for multicollinearity (Table 6; supplementary material). As a result, ‘retreat setting’ factor was found to be highly correlated with ‘shamanic framework’ and ‘live singing’ factors. As ‘shamanic framework’ and ‘live singing’ are less strongly correlated with each other, we assumed that they might both be components of ‘retreat setting’ factor; thus, we decided to exclude ‘retreat setting’ from further use as a covariate to avoid multicollinearity problems. Remaining variables (‘party setting’, ‘shamanic framework’, ‘live singing’, ‘disruptions’) were classified as confounding factors and included in the final model.

### The effect of cannabis on the subjective psychedelic experience

As represented by Table 3, Pillai’s trace value for cannabis was 0.097 (*p* = 0.009), confirming its relevance in the final model. This result further shows that cannabis use interacted with psychedelic-use in terms of its effects on subjective experience. The exact parameter estimates obtained from the MANCOVA are represented in Table 4, while the pair-wise comparisons after Bonferroni correction are in Table 7 (supplementary material). In order to help contextualise the parameter estimates, raw mean scores together with standard deviations for different psychedelic (Table 10; supplementary material) and cannabis conditions were also included (Table 11; supplementary material).

**Table 3:** MANCOVA results

Effect	Value	*F*	Hypothesis df	Error df	Sig
Cannabis	0.097	2.078	15	933	0.009 **
Party	0.060	3.938b	5	309	0.002 **
Shamanic	0.037	2.377b	5	309	0.039 *
Singing	0.026	1.674b	5	309	0.141
Disruptions	0.028	1.797b	5	309	0.113

The represented values are Pillai’s trace values, *F* values, hypothesis degrees of freedom, error degrees of freedom and sign. The statistic is an upper bound on *F* that yields a lower bound on the significance level

**Table 4:** Parameter estimates of the dependent variables in the MANCOVA model

DV	Parameter	*B*	Std. error	*t*	Sig	95% confid. interval		Part eta squared
						Lower	Upper	
MEQ	Cannabis: low dose	0.158	0.142	1.116	0.265	− 0.121	0.437	0.004
	Cannabis: med. dose	0.148	0.151	0.984	0.326	− 0.148	0.445	0.003
	Cannabis: high dose	0.484	0.193	2.507	0.013 *	0.104	0.864	0.02
	Party	− 0.153	0.054	− 2.812	0.005 **	− 0.26	− 0.046	0.025
	Shamanic	0.133	0.056	2.374	0.018 *	0.023	0.243	0.018
	Singing	0.109	0.054	2	0.046	0.002	0.216	0.013
	Disruptions	− 0.021	0.053	− 0.404	0.686	− 0.125	0.083	0.001
ASC_Vis	Cannabis: low dose	0.006	0.097	0.058	0.954	− 0.185	0.196	0
	Cannabis: med. dose	0.112	0.103	1.086	0.278	− 0.091	0.316	0.004
	Cannabis: high dose	0.4	0.132	3.022	0.003 **	0.139	0.66	0.028
	Party	0.001	0.037	0.036	0.971	− 0.072	0.075	0
	Shamanic	0.071	0.038	1.847	0.066	− 0.005	0.146	0.011
	Singing	0.106	0.037	2.837	0.005 **	0.032	0.179	0.025
	Disruptions	− 0.068	0.036	− 1.867	0.063	− 0.139	0.004	0.011
CEQ	Cannabis: low dose	− 0.311	0.114	− 2.734	0.007 **	− 0.535	− 0.087	0.023
	Cannabis: med. dose	− 0.137	0.121	− 1.134	0.258	− 0.376	0.101	0.004
	Cannabis: high dose	0.09	0.155	0.58	0.563	− 0.215	0.395	0.001
	Party	− 0.086	0.044	− 1.974	0.049 *	− 0.172	0	0.012
	Shamanic	0.084	0.045	1.865	0.063	− 0.005	0.172	0.011
	Singing	0.034	0.044	0.77	0.442	− 0.052	0.12	0.002
	Disruptions	0.036	0.043	0.855	0.393	− 0.047	0.12	0.002
EDI	Cannabis: low dose	− 0.001	0.032	− 0.019	0.985	− 0.063	0.061	0
	Cannabis: med. dose	0.035	0.034	1.029	0.304	− 0.032	0.101	0.003
	Cannabis: high dose	0.126	0.043	2.918	0.004 **	0.041	0.21	0.026
	Party	− 0.023	0.012	− 1.857	0.064	− 0.046	0.001	0.011
	Shamanic	0.015	0.012	1.221	0.223	− 0.009	0.04	0.005
	Singing	0.022	0.012	1.818	0.070	− 0.002	0.046	0.01
	Disruptions	− 0.017	0.012	− 1.477	0.141	− 0.041	0.006	0.007
EBI	Cannabis: low dose	− 0.014	0.152	− 0.095	0.925	− 0.313	0.284	0
	Cannabis: med. dose	0.154	0.162	0.954	0.341	− 0.164	0.472	0.003
	Cannabis: high dose	0.073	0.207	0.354	0.724	− 0.334	0.48	0
	Party	− 0.188	0.058	− 3.223	0.001 **	− 0.303	− 0.073	0.032
	Shamanic	0.169	0.06	2.822	0.005 **	0.051	0.287	0.025
	Singing	0.088	0.058	1.518	0.130	− 0.026	0.203	0.007
	Disruptions	− 0.021	0.057	− 0.375	0.708	− 0.133	0.09	0

The represented values are beta values (*B*) which represent the differences between the average scores of the reference group and group of interest, together with their significant levels (*p*) informing of the significance of these findings. (* = *p* < 0.05, ** = *p* < 0.01, *** = *p* < 0.001). *DV* dependent variable, *Med*. medium, *MEQ* Mystical Experience Questionnaire, *ASC-Vis* Altered States of Consciousness visual subscales, CEQ Challenging Experience Questionnaire, *EDI* Ego Dissolution Inventory, *EBI* Emotional Breakthrough Inventory

**Fig. 4:**
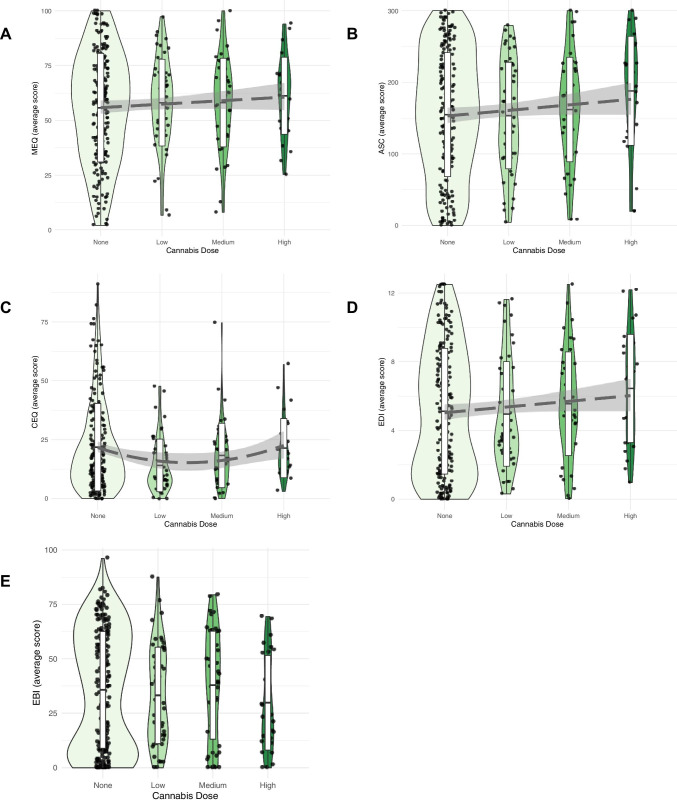
Results of regression modelling conducted for cannabis doses (none: *n* = 195, low: *n* = 53, medium: *n* = 45 or high: *n* = 28) taken alongside of a psychedelic drug. Analysis conducted for the following questionnaires: **A** Mystical Experience Questionnaire (MEQ), **B** Altered States of Consciousness–Visual Subscales (ASC-Vis), **C** Challenging Experience Questionnaire (CEQ), **D** Ego Dissolution Inventory (EDI), **E** Emotional Breakthrough Inventory (EBI). The width of the violin plots is proportional to the number of samples

Regression modelling results (Fig. 4) show that the presence of cannabis significantly altered the quality of a psychedelic experience in several dimensions. Detailed estimates for each variable, together with standard error (S.E.) and *p* values for both cannabis and the confounding factors in each questionnaire are represented in Table 5. Note that only the models with the lowest BIC (among models considering linear, quadratic and cubic relationship on cannabis dose) were reported.

**Table 5:** Parameter estimates of the dependent variables in the regression modelling

Outcome	Variable	Estimate	S.E	*p* value
MEQ	Cannabis dose	3.12	1.28	0.015 *
	Party	− 8.60	3.20	0.008 **
	Shamanic	7.72	3.19	0.016 *
	Singing	6.07	3.09	0.050 *
	Disruption	− 1.00	2.89	0.730
ASC-Vis	Cannabis dose	12.65	4.67	0.007 **
	Party	1.51	11.68	0.898
	Shamanic	20.98	11.65	0.073
	Singing	31.33	11.27	0.006 **
	Disruption	− 18.39	10.56	0.083
CEQ	Cannabis dose	− 8.82	3.05	0.004 **
	Cannabis dose^2	3.28	1.15	0.004 **
	Party	− 5.20	2.38	0.030 *
	Shamanic	4.30	2.37	0.071
	Singing	1.82	2.30	0.43
	Disruption	1.80	2.16	0.406
EDI	Cannabis dose	0.51	0.20	0.010 *
	Party	− 0.89	0.49	0.072
	Shamanic	0.58	0.49	0.243
	Singing	0.84	0.47	0.079
	Disruption	− 0.60	0.45	0.177
EBI	Cannabis dose	1.07	1.41	0.447
	Party	− 12.07	3.52	< .001 ***
	Shamanic	9.82	3.51	0.005 **
	Singing	5.24	3.40	0.124
	Disruption	− 1.28	3.18	0.689

The represented values show parameter estimates together with their standard errors (S.E.) and *p* values

*MEQ* Mystical Experience Questionnaire, *ASC-Vis* Altered States of Consciousness visual subscales, *CEQ* Challenging Experience Questionnaire, *EDI* Ego Dissolution Inventory, *EBI* Emotional Breakthrough Inventory

A positive linear relationship with increasing doses of cannabis was recorded for the MEQ (*p* = 0.015), ASC-Vis (*p* = 0.007) and EDI (*p* = 0.010). This means that higher doses of cannabis taken alongside the psychedelic drug resulted in higher average scores obtained in these questionnaires. The CEQ (*p* = 0.004 **) follows a quadratic trend, with low cannabis dose being associated with lower CEQ scores, whereas higher doses were linked to increased CEQ scores. No relationship was found for EBI; however, it should be noted that despite the mean levels staying at a similar level, the top range of obtained scores was lower with increasing doses of cannabis; meaning that few participants who took higher doses of cannabis also obtained high scores on the EBI. A similar phenomenon was also observed for MEQ, but with compression of the bottom range of scores with increasing doses; meaning that participants were less likely to have very low MEQ scores if taking high doses of cannabis.

### Role of cannabis in modulating challenging experiences

**Fig. 5:**
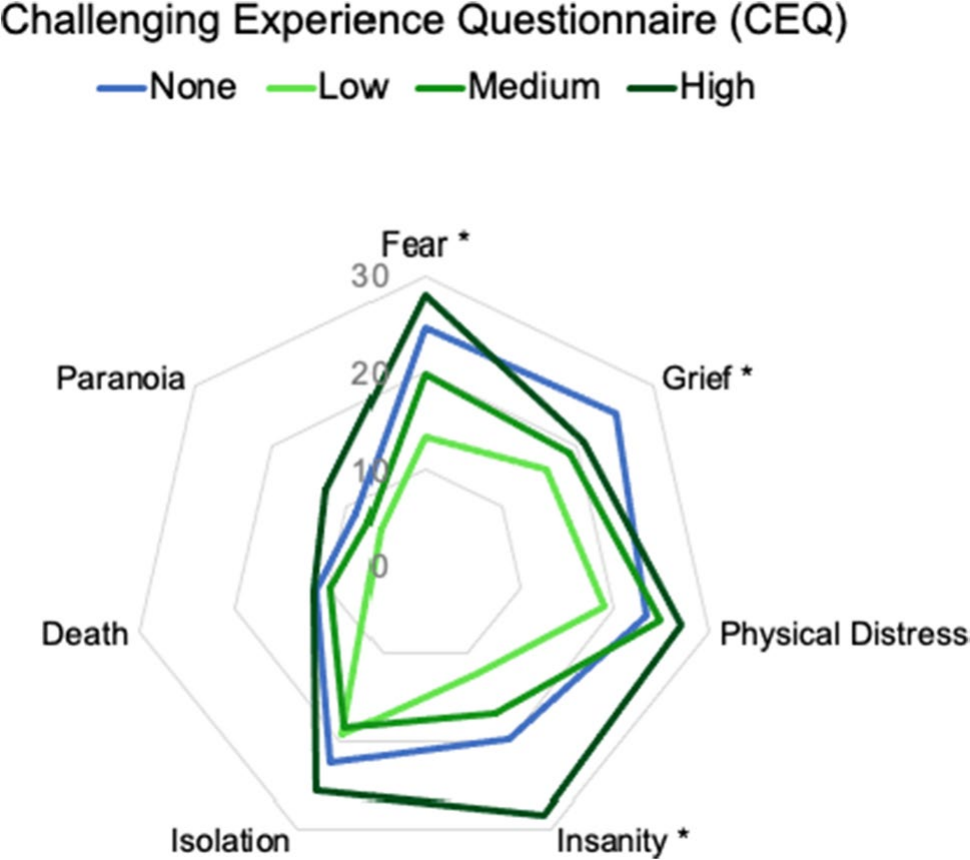
Effect of cannabis dose (none, low, medium, high) taken concomitantly with a classic psychedelic on the various dimensions of the Challenging Experience Questionnaire. The range on CEQ is 0–130; however, 0–30 was represented on the graph for better clarity of differences between groups. All participants (*n* = 321) took a serotonergic psychedelic (LSD, psilocybin and others with a similar mode of action), and are grouped based on whether they also used cannabis during their experience, with *n* = 195 having used none, *n* = 53 a low dose, *n* = 45 a medium dose and *n* = 28 a high dose of cannabis. Estimated marginal means were adjusted for other variables appearing in the model (party setting, shamanic framework, live singing and disruptions). Significance asterisks represent results of a univariate analysis, with * = *p* < 0.05

The dose-dependent effect of cannabis on the various dimensions of CEQ during a psychedelic experience is illustrated by Fig. 5. While the overall CEQ score follows a quadratic trend (as represented by Fig. 4C), univariate analyses of the subscales show that only in certain subscales the effect is significant. The subscales for which the effect is significant are the following: Fear, Grief and Insanity. In both Fear and Insanity subscales, the lowest CEQ score was obtained by group using a low dose of cannabis, followed by medium dose of cannabis, followed by no cannabis, and with the highest score recorded for those using a high dose of cannabis. The same trend is also seen for Paranoia, and Death subscales of the CEQ; however, the results do not cross the significance threshold. In Grief subscale, the lowest score was recorded for the group using low dose of cannabis, followed by medium, high, and with the highest score obtained by group which used no cannabis. For most of the subscales (except for Isolation), the lowest score was recorded in the group using a low cannabis dose, and for most of the subscales (except for Grief), the highest score was recorded for the high cannabis dose group. Please see the Appendix 2 for specific items composing each of the subscales, and Fig. 6 (supplementary material) for a representation of MEQ and EDI subscales. Please also remember that all of these are used alongside a classical psychedelic (see the ‘3’ section).

## Discussion

The current study investigated the effects of concomitant cannabis use on the subjective quality of a psychedelic experience across a spectrum of measures including mystical type, visual, challenging, ego-dissolution and emotional breakthrough domains. We found 39% of participants in the current study used psychedelics together with cannabis, supporting findings also reported by Licht et al. (2012), where a third of survey participants admitted using cannabis together with LSD or psilocybin, either often or always.

We found evidence of more intense mystical-type, ego dissolution and visual experiences in conjunction with cannabis use, as a linearly as a function of cannabis dose. A quadratic relationship was found for challenging experiences, indicating less challenging experiences with low dose cannabis but more challenging experiences with higher doses. No relationship was found between cannabis and experiences of emotional breakthrough. We are mindful not to be too hasty in inferring that cannabis use directly caused these effects and thus will explore different explanations for our findings, including, first, the possibility that cannabis may indeed have had a direct effect.

That the presence of cannabis tended to be associated with more intense psychedelic effects may be because cannabis itself induces subjective effects that are similar to some effects of psychedelics, such as euphoria, changes in perception of time, intensification of sensory perception and hyper-associative thinking (Tart 1970; Adamec et al. 1976; Barrett et al. 2018). Indeed, cannabis is sometimes classified as an ‘atypical psychedelic’ (Garcia-Romeu et al. 2016), or ‘psychedelic-like’ agent (Szabó et al. 2015). Furthermore, recent studies have shown that the THC blood levels correlate positively with higher scores on 5-Dimensional Altered States of Consciousness Rating Scale (5D-ASC) (Zaytseva et al. 2019)—a scale that is often used to assess the subjective effects of psychedelics. As with classic psychedelics (Byock 2018; Carhart-Harris et al. 2018b), the subjective action of cannabis can be dependent on the cultural context in which the drug is used (Adamec et al. 1976; Pihl et al. 1979), and varies both across and within individuals (Atakan et al. 2012; Zaytseva et al. 2019) and with the context in which the drug is used (Tart 1970). We attempted to control for these elements in the current study by asking participants specific questions which covered a broad spectrum of factors (‘set’ and ‘setting’, personality structure, mental health history and others) and include those we found relevant as covariates in our final analysis. Although we attempted to assess the cultural context by asking questions about whether the experience took place in a retreat setting or within a shamanic guiding framework, we wish to acknowledge that the study participants were not of specific religious practices, like Santo Daime, who use ayahuasca together with cannabis during religious ceremonies (MacRae 1998), or other specific indigenous groups, which might in consequence limit the implications of our study to practices outside of the mentioned contexts.

While the molecular basis of the synergistic effects between cannabis and psychedelics was not explored in the current study, the obtained results are suggestive of its existence. Despite serotonergic psychedelics and cannabis not inducing cross-tolerance (Isbell and Jasinski 1969) and having seemingly different modes of action, recent studies have brought to attention a potential degree of overlap in receptor targets of both of these drug classes. In rodents, both the lack of 5-HT_2A_R and use of 5-HT_2A_R antagonist limited the THC-induced cognitive impairment, which is indicative of a functional interaction between the two receptors (Viñals et al. 2015). In addition, the other constituent of cannabis, CBD, has been reported to facilitate 5-H_1A_ receptor-mediated neurotransmission (Resstel et al. 2009) and in high doses act as an inverse agonist of 5-HT_1A_R (Martínez-Aguirre et al. 2020). In human brain, the majority of 5-HT_1A_R are located postsynatpically and have a largely inhibitory function over 5-HT_2A_Rs (Carhart-Harris and Nutt 2017); therefore, an inverse agonist action on this receptor would result in an increase in the serotonergic transmission. Indeed, it has previously been suggested that also endocannabinoids modulate the serotonin system and play a key role in the regulation of brain excitability (Haj-Dahmane and Shen 2011). Other studies reveal increased synaptic 5-HT concentration as a response to CB1 stimulation (Burokas et al. 2014). However, whether increased 5-HT would function synergistically with psychedelics known to directly stimulate the 5-HT_2A_ receptor (Nichols 2000) has not been addressed yet.

### The action of cannabis: anxiolytic and anxiogenic

The quadratic relationship between cannabis dose and challenging experiences fits with the notion that cannabis can exert differential, including potentially opposing effects, depending on dose (and perhaps potency)—i.e., low doses may be anxiolytic and high doses, anxiogenic. In fact, this two-way pattern has also previously been reported in animal work, suggesting that at low doses, cannabis may act as an anti-depressant, while high doses may worsen depression (Bambico et al. 2007). Cannabinoids are licensed for the treatment of side effects due to cancer chemotherapy, or neurological symptoms, where anxiety relief is desirable (Turna et al. 2017). Cannabis is increasingly used to cope with anxiety, stress or insomnia in patients suffering from post-traumatic stress disorder (PTSD) (Yarnell 2015). The ratio of THC to CBD in cannabis products predicts whether cannabis is more likely to induce anxiogenic- or -lytic effects (Kamal et al. 2018) with anxiolytic qualities largely induced by CBD, the nonintoxicating constituent of cannabis (Iffland and Grotenhermen 2017). It may be there is an optimal ratio of CBD to THC in cannabis that renders mystical-type and ego dissolution experiences more likely, without also intensifying challenging experiences. Alternatively, a high dose of CBD on its own may also be capable of increasing activity of 5-HT_2A_Rs through its inverse agonist action on 5-HT_1A_R (Martínez-Aguirre et al. 2020) without carrying the negative effects of THC.

Assessing the sub-factors of the CEQ, perceived grief was reduced for all of the reported cannabis doses, which suggests its capacity to potentially limit the experiential acceptance, considered one of the key mechanisms of action in psychedelic-assisted psychotherapy for depression (Watts et al. 2017). We should also note that a high dose of cannabis resulted in increased scores on the fear subscale. This warrants caution in potential cannabis use in clinical settings, especially in vulnerable populations. Additionally, an association was found between high-dose cannabis and the insanity subscale of the CEQ in particular. It seems prudent to consider not just the prevalence (which may be low) but also the severity of (e.g. rare) negative responses to psychedelics, and acute psychotic or psychosis-like symptoms might be regarded in this way, i.e., one of the more severe possible responses to psychedelics or cannabis. This matter seems especially relevant given literature on the psychotomimetic effects of both cannabis and psychedelics (Carhart-Harris et al. 2013), as well as complex questions over the psychotogenic potential of cannabis use (Hamilton and Sumnall 2021). We hope that through reporting on the subjective effects of psychedelics use in combination with cannabis, we can contribute to raising awareness among recreational users, allowing them for better informed decision making; an especially important feature of harm reduction strategies in adolescents (Baltzer et al. 2008), who are the most susceptible group to experience negative effects of cannabis on their mental wellbeing (Gobbi et al. 2019).

## Limitations

Firstly, a limitation of this study is that we did not directly assess the doses of the psychedelic or cannabis used, relying instead on the subjective report describing the perceived quantity. To report the dose of a taken psychedelic, participants were asked to select a total dose of the drug used, standardised to LSD dose ranges (for details, see the ‘3’ section). As an improvement for further studies, different dose prompts for a broader range of drugs should be pre-encoded into the questionnaire. Similarly, to report the dose of cannabis, participants were not presented with suggested dose ranges and only asked to indicate whether they took what they considered a low, medium or high dose. We should acknowledge that this subjective assessment and report might be biased by various factors, including sample inaccuracy, poor inter-subject reliability or standardisation in assessment, previous drug experience and environmental/social factors. Indeed, it seems quite possible that what subjects reported as ‘high doses’, more accurately translates as ‘doses that caused particularly strong effects’. This discrepancy might have influenced the obtained results, specifically the dose-dependence of reported outcomes; thus, considerable caution must be exercised when interpreting the results.

The ordinal, non-continuous nature of the assessed cannabis dose variable furthermore forbids us from making strong conclusions about the exact nature of the relationships, considering the uncertain spacing between subjectively rated dose levels. Despite this limitation, the demonstrated inverse U-shape effect between subjective dose and CEQ scores remains valid, even if the precise curvature of the U-shape might be different based on actual dosage.

Having a precise specification of the time when cannabis was taken (e.g. at the very beginning of the psychedelic experience, or at its ‘peak’) would improve our understanding of effects, as well as the potential direction of causality i.e., was the cannabis use a cause or consequence of some specific effects or experience? A previous study reported that many people use cannabis throughout the psychedelic session, but that some people also use it before or after (Licht et al. 2012). It is plausible that taking cannabis at the peak of the psychedelic experience could increase and/or prolong mystical-type or ‘ego-dissolution’ experiences, while low doses at the very beginning of the experience might reduce some anxiety related to the onset of psychedelic effects. Additionally, it would be informative to capture the route of cannabis administration as this affects its time course (Barrus et al. 2016). Another limitation of the study which needs to be mentioned is the lack of collected history of lifetime and recent cannabis and psychedelic usage. More experienced users may experience different effects from the drug; frequent use of LSD or psilocybin may lead to tolerance, similarly to cannabis where recurrent use reinforces minimisation of negative effects, such as cognitive impairment due to downregulation of CB1 receptors (Ceccarini et al. 2015). Moreover, given that chronic exposure to cannabis may promote a pro-hallucinogenic confirmation of 5-HT_2A_R (Ibarra-Lecue et al. 2018), it would be interesting to evaluate drug use history as a potential covariate in future studies.

Finally, we are cognisant that our study design prevents us from making inferences on the causal effects of cannabis use on subjective experience. We would also like to note that the effect sizes are overall small; therefore, the key constituents of the subjective experience cannot be attributed directly to cannabis but rather modified by it. Additionally, while in Bonferroni adjustment (Table 7; supplementary material), the significance effect for MEQ disappears, this should not be interpreted as a key result of the current study as Bonferroni test often fails to detect real differences and contributes to type II errors (Lee and Lee 2018), thus does not invalidate the reported results.

It is quite plausible that some individuals may use cannabis in an attempt to alter effects or experiences principally induced by the psychedelic, in the same way that some cannabis users report using cannabis to ‘self-medicate’ for psychiatric symptoms. Future controlled research is needed to better assess causal interactions between cannabis and psychedelics in relation to acute and more enduring psychological effects.

## Conclusions

Overall, this study provided a first quantitative insight into the modulation of subjective psychedelic effects by cannabis. Concomitant cannabis consumption was dose-dependently associated with higher scores of mystical-type experience, ego-dissolution and visual alterations. Cannabis use was also found to relate to challenging aspects of the psychedelic experience but not in a conventional linear way, i.e. low doses were associated with lower CEQ scores, whereas high doses were associated with higher scores, and the ‘insanity’ sub-scale in particular.

Given the high rates of cannabis use in concert with the use of psychedelic substances, the current research has important implications for harm reduction education but may, eventually, also have implications for therapeutic use, considering that some of the therapeutically desirable psychological effects associated with psychedelics may, in theory, be enhanced by concomitant cannabis use.

## Supplementary Information

Below is the link to the electronic supplementary material.

Supplementary file1 (DOCX 80 KB)

## Abbreviations

1P-LSD: 1-Propionyl-lysergic acid diethylamide
2C-B: 2,5-Dimethoxy-4-bromophenethylamine
2C-T-21: 4-(2-Fluoroethylthio)-2,5-dimethoxyphenethylamine
4-AcO-DMT: O-Acetylpsilocin
4-Ho-MET: 4-Hydroxy-*N*-methyl-*N*-ethyltryptamine
5-HT: Serotonin
5-HT_1A_R: Serotonin 1A receptor
5-HT_2A_R: Serotonin 2A receptor
5-MeO-DMT: 5-Methoxy-*N*,*N*-dimethyltryptamine
5D-ASC: 5-Dimensional Altered States of Consciousness Rating Scale
AL-LAD: 6-Allyl-6-nor-LSD
ASC-Vis: Visual subscales of the Altered States of Consciousness Rating Scale
CB1: Cannabinoid receptor type 1
CEQ: Challenging Experience Questionnaire
DMT: *N*,*N*-Dimethyltryptamine
EBI: Emotional Breakthrough Inventory
EDI: Ego Dissolution Inventory
LSD: Lysergic acid diethylamide
MANCOVA: Multivariate analysis of covariance
MEQ: Mystical Experience Questionnaire
OCD: Obsessive–compulsive disorder
PTSD: Post-traumatic stress disorder
THC: Tetrahydrocannabinol
VAS: Visual analogue scale
BIC: Bayesian Information Criterion

## Footnotes

1 The data presented in this paper is part of a large prospective study, see Haijen et al. (2018). The current paper only describes the elements of the design and data that are relevant to the current study.
