# Supplementary material for: Psychedelic experience dose-dependently modulated by cannabis: results of a prospective online survey
Source: Psychopharmacology (Berl). 2021 Nov 4;239(5):1425–40. doi: 10.1007/s00213-021-05999-1 (PMC9110465; doi:10.1007/s00213-021-05999-1)
Supplement: Supplementary file 1 — Supplementary file1 (DOCX 80 KB) [file 213_2021_5999_MOESM1_ESM.docx]

# SUPPLEMENTARY MATERIAL (SI)

## Selection of confounding factors

| **Table 6.** Correlations between covariates shown to have a significantly different distribution across different conditions of cannabis use (none, low, medium, and high dose) together with a psychedelic. | | | | | |
| --- | --- | --- | --- | --- | --- |
|  | **Retreat** | **Party** | **Shamanic** | **Singing** | **Disruptions** |
| **Retreat** |  | -.205** | .374** | .458** | -0.003 |
| **Party** | -.205** |  | -.227** | -0.078 | 0.1 |
| **Shamanic** | .374** | -.227** |  | .330** | -0.016 |
| **Singing** | .458** | -0.078 | .330** |  | 0.054 |
| **Disruptions** | -0.003 | 0.1 | -0.016 | 0.054 |  |
| *Values indicate Pearson Correlation values.*  *** Correlation is significant at the 0.01 level (2-tailed).* | | | | | |

## Demographics

Most participants were based in North America, Europe, and Australia. The majority of participants held university qualifications (54.5%) and were either in full-time employment (35.2%) or students (32.1%). More than half of the participants had no history of psychiatric illnesses (63.9%); among those who had a psychiatric diagnosis in their lifetimes, equally most common diagnoses were: anxiety disorder (16.5%) and major depressive disorder (16.5%).

## Detailed statistical analyses

| **Table 7.** Pairwise Comparisons after Bonferroni correction. | | | | | | | |
| --- | --- | --- | --- | --- | --- | --- | --- |
| DV | (I) Cannabis dose | (J) Cannabis dose | Mean diff (I-J) | Std. Error | Sig. | 95% Confidence Interval for Difference | |
|  |  |  |  |  |  | Lower | Upper |
| **MEQ** | High | Medium | 0.336 | 0.224 | 0.804 | -0.258 | 0.929 |
|  |  | Low | 0.326 | 0.217 | 0.798 | -0.249 | 0.901 |
|  |  | None | 0.484 | 0.193 | 0.076 | -0.029 | 0.997 |
|  | Medium | High | -0.336 | 0.224 | 0.804 | -0.929 | 0.258 |
|  |  | Low | -0.01 | 0.185 | 1 | -0.502 | 0.483 |
|  |  | None | 0.148 | 0.151 | 1 | -0.252 | 0.549 |
|  | Low | High | -0.326 | 0.217 | 0.798 | -0.901 | 0.249 |
|  |  | Medium | 0.01 | 0.185 | 1 | -0.483 | 0.502 |
|  |  | None | 0.158 | 0.142 | 1 | -0.218 | 0.534 |
|  | None | High | -0.484 | 0.193 | 0.076 | -0.997 | 0.029 |
|  |  | Medium | -0.148 | 0.151 | 1 | -0.549 | 0.252 |
|  |  | Low | -0.158 | 0.142 | 1 | -0.534 | 0.218 |
| **ASC_Vis** | High | Medium | 0.288 | 0.153 | 0.368 | -0.119 | 0.694 |
|  |  | Low | .394* | 0.148 | 0.05 | 0 | 0.788 |
|  |  | None | .400* | 0.132 | 0.016 * | 0.048 | 0.751 |
|  | Medium | High | -0.288 | 0.153 | 0.368 | -0.694 | 0.119 |
|  |  | Low | 0.107 | 0.127 | 1 | -0.231 | 0.444 |
|  |  | None | 0.112 | 0.103 | 1 | -0.162 | 0.387 |
|  | Low | High | -.394* | 0.148 | 0.05 | -0.788 | 0 |
|  |  | Medium | -0.107 | 0.127 | 1 | -0.444 | 0.231 |
|  |  | None | 0.006 | 0.097 | 1 | -0.252 | 0.263 |
|  | None | High | -.400* | 0.132 | 0.016 * | -0.751 | -0.048 |
|  |  | Medium | -0.112 | 0.103 | 1 | -0.387 | 0.162 |
|  |  | Low | -0.006 | 0.097 | 1 | -0.263 | 0.252 |
| **CEQ** | High | Medium | 0.227 | 0.180 | 1 | -0.25 | 0.704 |
|  |  | Low | 0.401 | 0.174 | 0.131 | -0.061 | 0.863 |
|  |  | None | 0.09 | 0.155 | 1 | -0.322 | 0.502 |
|  | Medium | High | -0.227 | 0.180 | 1 | -0.704 | 0.25 |
|  |  | Low | 0.174 | 0.149 | 1 | -0.222 | 0.569 |
|  |  | None | -0.137 | 0.121 | 1 | -0.459 | 0.184 |
|  | Low | High | -0.401 | 0.174 | 0.131 | -0.863 | 0.061 |
|  |  | Medium | -0.174 | 0.149 | 1 | -0.569 | 0.222 |
|  |  | None | -.311* | 0.114 | 0.04 * | -0.613 | -0.009 |
|  | None | High | -0.09 | 0.155 | 1 | -0.502 | 0.322 |
|  |  | Medium | 0.137 | 0.121 | 1 | -0.184 | 0.459 |
|  |  | Low | .311* | 0.114 | 0.04 | 0.009 | 0.613 |
| **EDI** | High | Medium | 0.091 | 0.050 | 0.412 | -0.041 | 0.223 |
|  |  | Low | 0.126 | 0.048 | 0.056 | -0.002 | 0.254 |
|  |  | None | .126* | 0.043 | 0.023 * | 0.011 | 0.24 |
|  | Medium | High | -0.091 | 0.050 | 0.412 | -0.223 | 0.041 |
|  |  | Low | 0.035 | 0.041 | 1 | -0.075 | 0.145 |
|  |  | None | 0.035 | 0.034 | 1 | -0.055 | 0.124 |
|  | Low | High | -0.126 | 0.048 | 0.056 | -0.254 | 0.002 |
|  |  | Medium | -0.035 | 0.041 | 1 | -0.145 | 0.075 |
|  |  | None | -0.001 | 0.032 | 1 | -0.084 | 0.083 |
|  | None | High | -.126* | 0.043 | 0.023 * | -0.24 | -0.011 |
|  |  | Medium | -0.035 | 0.034 | 1 | -0.124 | 0.055 |
|  |  | Low | 0.001 | 0.032 | 1 | -0.083 | 0.084 |
| **EBI** | High | Medium | -0.081 | 0.239 | 1 | -0.717 | 0.555 |
|  |  | Low | 0.087 | 0.232 | 1 | -0.528 | 0.703 |
|  |  | None | 0.073 | 0.207 | 1 | -0.476 | 0.622 |
|  | Medium | High | 0.081 | 0.239 | 1 | -0.555 | 0.717 |
|  |  | Low | 0.168 | 0.199 | 1 | -0.359 | 0.696 |
|  |  | None | 0.154 | 0.162 | 1 | -0.275 | 0.583 |
|  | Low | High | -0.087 | 0.232 | 1 | -0.703 | 0.528 |
|  |  | Medium | -0.168 | 0.199 | 1 | -0.696 | 0.359 |
|  |  | None | -0.014 | 0.152 | 1 | -0.417 | 0.388 |
|  | None | High | -0.073 | 0.207 | 1 | -0.622 | 0.476 |
|  |  | Medium | -0.154 | 0.162 | 1 | -0.583 | 0.275 |
|  |  | Low | 0.014 | 0.152 | 1 | -0.388 | 0.417 |
| *DV = Dependent variable* | | | | | | | |

## The effect of cannabis on separate subscales

| **A** | **B** |
| --- | --- |
|  |  |

**Fig 6.** The dose-dependent effect of cannabis on various dimensions of psychedelic experience. All participants (n=321) took a serotonergic psychedelic (LSD, psilocybin, and others with a similar mode of action), and are grouped based on whether they also used cannabis during their experience, with n=195 having used none, n=53 a low dose, n=45 a medium dose, and n=28 a high dose of cannabis. Estimated marginal means were calculated and plotted on radar chart according to the score on stated dimensions of each questionnaire. The values were adjusted for other variables appearing in the model (party setting, shamanic framework, live singing, and disruptions), consistent throughout the current work. The significance asterisks represent results of a univariate analysis, with * = p<0.05 (A) Estimated marginal means for the Mystical Experience Questionnaire (MEQ) dimensions: mystical*, positive mood*, ineffability, transcendence of time and space. (B) Estimated marginal means for the visual subscales of Altered States of Consciousness (ASC-Vis) questionnaire dimensions: complex imagery, elementary imagery*, and audio-visual synaesthesia.

| **Table 8.** Univariate (ANOVA) analysis of separate questionnaire subscales for Challenging Experience Questionnaire (CEQ), Mystical Experience Questionnaire (MEQ), and visual subscales of Altered States of Consciousness Questionnaire. All participants have used serotonergic psychedelics and cannabis dose (none=0, low=1, medium=2, high=3) was used as a fixed factor. | | | | |
| --- | --- | --- | --- | --- |
|  |  | F | Sig | Partial Eta Squared |
| **CEQ** | Fear | 3.818 | .010 * | .035 |
|  | Grief | 2.701 | .046 * | .025 |
|  | Physical Distress | 1.481 | .220 | .014 |
|  | Insanity | 3.085 | .028 * | .029 |
|  | Isolation | .685 | .562 | .007 |
|  | Death | .954 | .415 | .009 |
|  | Paranoia | 1.472 | .222 | .014 |
| **MEQ** | Mystical | 3.220 | .023 * | .030 |
|  | Positive | 2.660 | .048 * | .025 |
|  | Space Time | .399 | .754 | .004 |
|  | Ineffability | .749 | .523 | .007 |
| **ASC-Vis** | Complex Imagery | 2.544 | .056 | .024 |
|  | Elementary Imagery | 3.063 | .028 * | .029 |
|  | Audio-Visual | 1.956 | .121 | .018 |
| ** = p < 0.05*  *** = p < 0.01* | | | | |

| **Table 9.** Polydrug use frequencies across different cannabis conditions. | | | | | |
| --- | --- | --- | --- | --- | --- |
|  | | **Cannabis** | | | |
|  |  | **None** | **Low** | **Medium** | **High** |
| **Alcohol** | **None** | 183 | 45 | 39 | 20 |
|  | **Low** | 9 | 6 | 4 | 6 |
|  | **Medium** | 3 | 2 | 1 | 1 |
|  | **High** | 0 | 0 | 1 | 1 |
| **Stimulants** | **None** | 194 | 52 | 42 | 27 |
|  | **Low** | 1 | 1 | 1 | 0 |
|  | **Medium** | 0 | 0 | 2 | 1 |
|  | **High** | 0 | 0 | 0 | 0 |
| **Tobacco** | **None** | 167 | 34 | 25 | 14 |
|  | **Low** | 13 | 15 | 14 | 6 |
|  | **Medium** | 13 | 3 | 5 | 5 |
|  | **High** | 2 | 1 | 0 | 0 |
| *The represented values are absolute frequencies representative of the number of participants.* | | | | | |

| **Table 10.** Raw scores of outcome measures by psychedelic dose. | | | | | | | | | |  | |  |
| --- | --- | --- | --- | --- | --- | --- | --- | --- | --- | --- | --- | --- |
| **Psychedelic dose** | | | | | | | | | | | | |
|  | low | SD | moder | SD | high | SD | v high | SD | ext high | | SD | |
| **MEQ** | 38.02 | 23.33 | 54.12 | 22.90 | 63.26 | 19.88 | 57.31 | 17.97 | 69.37 | | 21.79 | |
| **CEQ** | 13.25 | 13.51 | 17.95 | 15.48 | 22.22 | 17.20 | 25.48 | 17.77 | 22.35 | | 18.13 | |
| **EDI** | 3.44 | 3.32 | 4.73 | 3.30 | 6.09 | 3.20 | 5.16 | 3.50 | 7.46 | | 3.43 | |
| **EBI** | 23.36 | 22.04 | 34.13 | 26.25 | 37.94 | 23.75 | 35.73 | 25.54 | 41.07 | | 25.57 | |
| **ASC-Vis** | 77.39 | 82.67 | 147.61 | 82.59 | 174.62 | 69.68 | 193.15 | 60.49 | 205.54 | | 73.44 | |
| *The represented values are means and their corresponding standard deviations (SDs*).  moder = moderate, v high = very high, ext high = extremely high | | | | | | | | | | | | |

| **Table 11.** Raw scores of outcome measures by cannabis dose. | | | | | | | | |
| --- | --- | --- | --- | --- | --- | --- | --- | --- |
|  | **Cannabis dose** | | | | | | |  |
|  | none | SD | low | SD | med | SD | high | SD |
| **MEQ** | 56.04 | 24.66 | 58.10 | 19.60 | 58.12 | 19.99 | 61.24 | 17.29 |
| **CEQ** | 21.91 | 18.72 | 14.11 | 11.06 | 18.31 | 13.52 | 21.46 | 12.34 |
| EDI | 5.18 | 3.66 | 4.96 | 3.01 | 5.55 | 2.98 | 6.44 | 3.09 |
| **EBI** | 35.66 | 26.58 | 33.17 | 21.89 | 37.86 | 24.51 | 29.81 | 21.32 |
| **ASC-Vis** | 155.05 | 86.53 | 153.48 | 73.60 | 161.88 | 72.13 | 188.02 | 74.79 |
| *The represented values are means and their corresponding standard deviations (SDs*). | | | | | | | | |
